# A fuzzy-optimized hybrid ensemble model for yield prediction in maize-soybean intercropping system

**DOI:** 10.3389/fpls.2025.1567679

**Published:** 2025-05-22

**Authors:** Amna Ikram, Sunnia Ikram, El-Sayed M. El-kenawy, Adil Hussain, Amal H. Alharbi, Marwa M. Eid

**Affiliations:** ^1^ Department of Computer Science and IT, Government Sadiq College Women University, Bahawalpur, Pakistan; ^2^ Department of Software Engineering, The Islamia University, Bahawalpur, Pakistan; ^3^ School of ICT, Faculty of Engineering, Design and Information and Communication Technology (EDICT), Bahrain Polytechnic, Isa Town, Bahrain; ^4^ Applied Science Research Center. Applied Science Private University, Amman, Jordan; ^5^ School of Electronics and Control Engineering, Chang’an University, Xi’an, China; ^6^ Department of Computer Sciences, College of Computer and Information Sciences, Princess Nourah Bint Abdulrahman University, Riyadh, Saudi Arabia; ^7^ Faculty of Artificial Intelligence, Delta University for Science and Technology, Mansoura, Egypt; ^8^ Jadara University Research Center, Jadara University, Irbid, Jordan

**Keywords:** maize-soybean intercropping, yield prediction, fuzzy inference system, ensemble learning, genetic algorithm, random forest, CatBoost, ELM

## Abstract

Maize-soybean intercropping is a sustainable farming practice that optimizes resource use efficiency and improves yield potential. Accurate yield prediction is essential for effective agricultural management in such systems. This study proposes a Fuzzy-Optimized Hybrid Ensemble Model (FOHEM), integrating stacked ensemble machine learning algorithms with a fuzzy inference system (FIS) to improve yield prediction. The dataset includes four intercropping treatments: SM (sole maize), SS (sole soybean), 2M2S (two rows of maize with alternating two rows of soybean), and 2M3S (two rows of maize with alternating three rows of soybean). Key input features include environmental factors, soil nutrients, and management practices across different treatments. The FOHEM framework integrates the outputs of the FIS with a stacked ensemble model comprising Random Forest (RF), Categorical Boosting (CatBoost), and Extreme Learning Machine (ELM)). A genetic algorithm (GA) dynamically adjusts the weights between FIS and the ensemble model, optimizing final prediction while enhancing accuracy and robustness. Additionally, LIME and SHAP are used for model interpretability, and identifying yield influencing factors. The model is validated using performance metrics such as MSE, MAE, and R^2^. The results demonstrated that proposed model significantly enhances yield prediction accuracy, offering valuable insights for optimizing intercropping systems. This study highlights the potential of integrating machine learning, fuzzy inference and optimization techniques to advance precision agriculture and decision-making in sustainable farming.

## 1 Introduction

Agriculture serves as the foundation of global food production and plays a significant role in economic development. However, providing food security for a growing population while maximizing crop yield and maintaining environmental sustainability remains a significant challenge. Sustainable farming practices such as intercropping offer a promising solution by optimizing resource utilization, improving soil fertility, and enhancing overall yield stability (Toker et al., 2024). Additionally, agriculture contributes in strengthening adaptation and mitigation efforts against climate change (Bin Wu et al., 2014; Gao et al., 2020).

**Figure 1:**
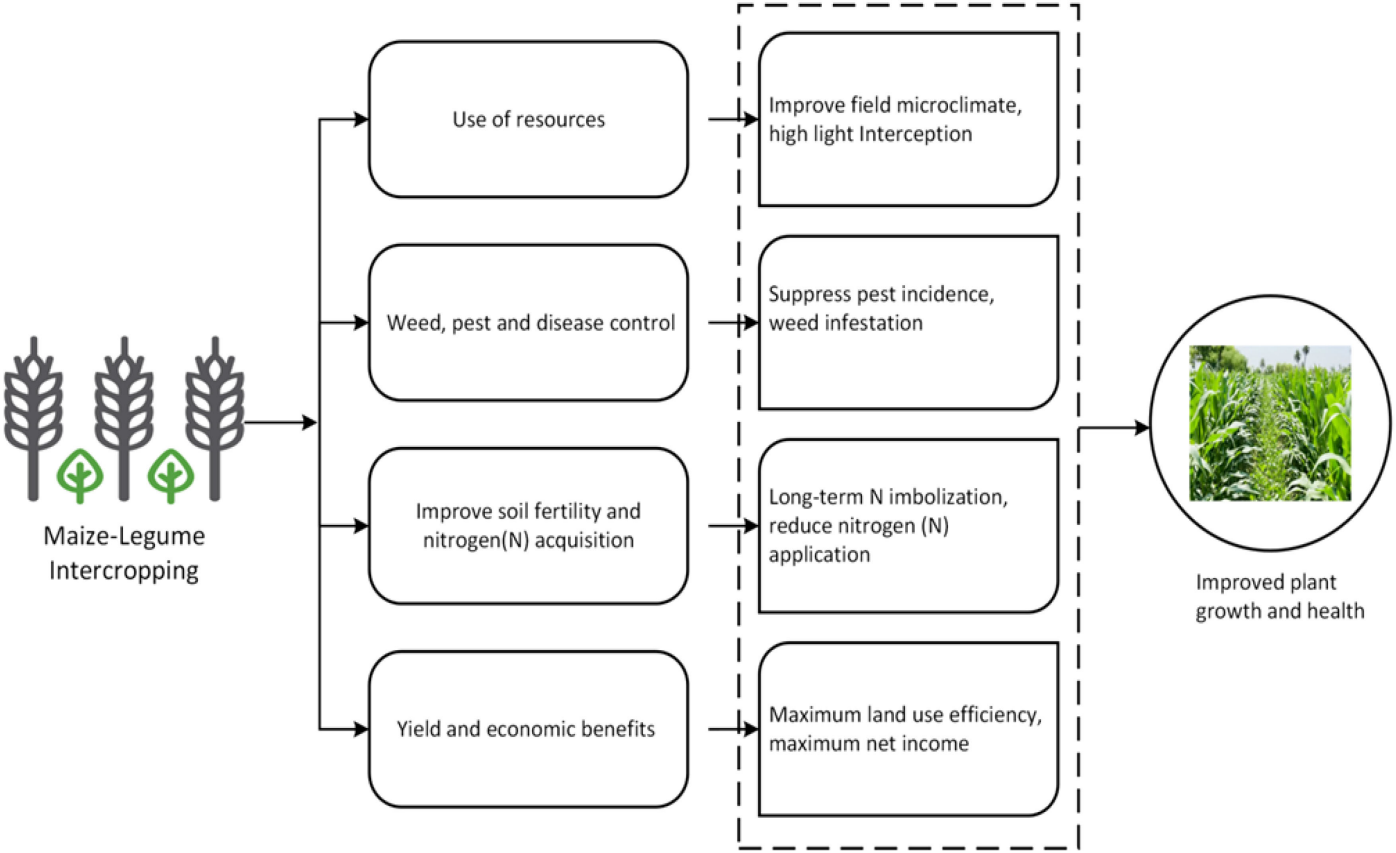
Maize-soybean intercropping is used to achieve crops, soil and environmental health (China-Pak Coop in Maize-Soybean Intercropping; Kumawat et al., 2022).

The existing monoculture agricultural system is one of the main causes of food insecurity in developing countries, which are low resilient to environmental and biotic pressures. According to recent research, countries with great number of crop varieties and crop groups have stronger inter-annual stability of total agricultural production (Jabbar et al., 2020). Intercropping is a sustainable agricultural technique where two or more crops are grown in the same field at the same time and is used to enhance crop productivity, improve soil fertility, and reduce environmental risks (Stomph et al., 2020; Raza et al., 2022). Among intercropping systems, maize-soybean intercropping is widely used due to its ability to enhance nitrogen fixation, optimize land use, and improve overall yield stability (see **Figure 1**). However, accurate yield prediction in such complex systems remains challenging due to interactions between crops, soil conditions, and climate variability.

Existing AI-driven yield prediction models have been extensively developed for monoculture systems, but there is a significant lack of research on their applicability to intercropping (see in **Table 1**). Although machine learning has significantly improved predictive accuracy in agriculture, most studies have focused on single crop systems, neglecting the complexities of intercropping. The limitations of existing models have led to several key research gaps, which are discussed in detail in section 2. These gaps include:

- Most predictive models are designed for monoculture, failing to address the crop interactions and resource competition inherent in intercropping.
- Existing machine learning models require extensive labeled data for training, but intercropping systems have complex, dynamic relationships that vary across regions, making it difficult to apply these models universally.
- Existing models mostly rely on static feature weights, assuming constant crop responses. In intercropping, species interactions vary dynamically due to changing nutrient uptake, root competition, and environmental influences, which static models cannot capture.
- Statistical models, such as regression-based approaches, struggle to adapt the nonlinear and dynamic nature of intercropping, while deep learning models often lack interpretability and adaptability, which are crucial in agronomic contexts.

**Table 1:** Summery of related work with research gap.

Study	Approach	Key Findings	Research Gap
(Wang et al., 2021)	Canopy modeling in intercropping	Optimized light use efficiency	Does not incorporate intercropping density variations for yield prediction.
(Keerthika et al., 2024)	Machine learning based soil classification	Suggested sole and intercrop combinations	Lacks real-time and dynamic yield prediction for intercropping.
(Atamanyuk et al., 2023)	Random sequence analysis	High accuracy but lacks adaptability	Does not integrate FIS to handle uncertainty in predictions.
(Azeem and Mai, 2024)	LAI-GDD modeling	Effective biomass prediction	Lacks hybrid machine learning integration for improved accuracy.
(Sadenova et al., 2021)	Statistical yield modeling	Strong correlation with official stats	Does not handle real-time yield variability under intercropping.
(Lobell and Burke, 2010)	Statistical *vs* process-based model	Panel models predict temperature effects well	Lacks soft computing and machine learning hybridization for dynamic yield.
(Jabed and Azmi Murad, 2024)	Machine learning for yield prediction	RF, SVR, and GBM improve accuracy	Does not incorporate uncertainty quantification techniques.
(Sharma et al., 2023)	CNN, RF, XGBoost for yield	RF performed well	Lacks model explainability and interpretability techniques.
(Abdel-salam et al., 2024).	Feature selection + optimized SVR	Higher efficiency	Does not integrate FIS for enhanced decision-making.
(Bhimavarapu et al., 2023)	IOF with LSTM	Reduced overfitting	Lacks soft computing integration for uncertainty handling.
(Upreti et al., 2024)	GA+ PSO for yield & pest prediction	Higher pest detection accuracy	Does not provide treatment specific yield predictions for intercropping.
(Elbasi et al., 2023)	Machine learning + IoT sensor data	High accuracy	Lacks explainability and interpretability for practical decision-making.

As highlighted in the literature review (**Table 1**), existing studies have primarily focused on yield prediction using standalone machine learning models or conventional statistical techniques. However, these approaches lack adaptability to effectively capture the dynamic crop interactions and environmental influences that are integral to intercropping systems.

**Figure 2:**
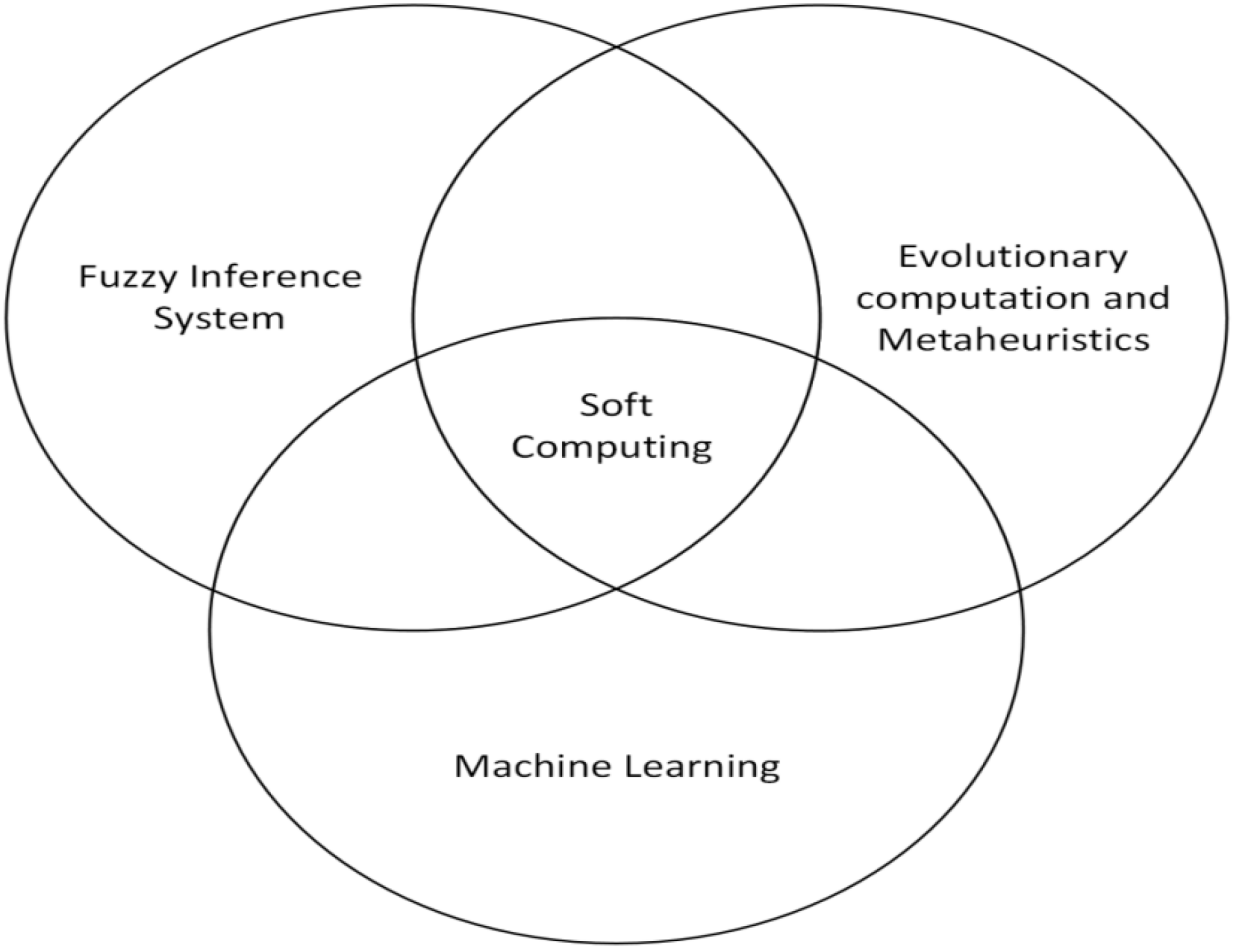
Soft computing framework for proposed system.

To address these challenges, this study proposes a fuzzy-optimized hybrid ensemble framework that combines fuzzy logic with stacked ensemble machine learning. A genetic algorithm is further employed to dynamically allocate weights between the fuzzy and machine learning components, improving predictive accuracy and robustness across different intercropping treatments (see **Figure 2**).

LIME and SHAP are also applied for model interpretability and transparency, enabling the identification of influential parameters such as total biomass, residue biomass and soil organic carbon, which provide valuable information for optimizing resource allocation. The dataset includes four distinct treatment groups based on density variations and planting patterns: SS (sole soybean), SM (sole maize), 2M2S (two rows of maize alternating with two rows of soybean), and 2M3S (two rows of maize alternating with three rows of soybean). Selected features encompass environmental practices pertinent to both sole and intercrop conditions, ensured the reliable predictions.

In proposed work, the research questions are investigated given below:

RQ1: How can integrating fuzzy inference system with machine learning improve the accuracy and reliability of yield prediction in intercropping systems compared to traditional agronomic models?
RQ2: How can computational models accurately predict yield for different intercropping treatments (SS, SM, 2M2S, and 2M3S) to support sustainable decision-making agriculture?
RQ3: How can optimization techniques (GA) and interpretability methods (LIME and SHAP) improve the accuracy, transparency, and adoption of AI-driven yield prediction models in precision agriculture?

Accurate yield prediction in intercropping is essential for optimizing input use and reducing economic risks. Traditional models lack adaptability required for complex cropping interactions, making soft computing and hybrid ensemble models valuable tools for agricultural decision-making.

The structure describes the literature review in section 2, methodology used in proposed work presented in section 3, Findings and Results of the model presented in section 4 and discussion, limitations, future work and conclusion are presented in section 5.

## 2 Literature review

Growing interest in sustainable agriculture has propelled intercropping to the forefront of research, with a marked increase in scientific studies dedicated to its potential. To effectively evaluate productivity and resource use efficiency within these systems, a robust conceptual framework is essential (Stomph et al., 2020). The accurate prediction of yield remains essential for optimizing intercropping because it reveals essential relationships between crop species and environmental conditions and management strategies. Predictive research models help identify essential factors like light interception, nutrient dynamics and planting density, enable scientists to create better resource management strategies. The development of reliable yield prediction system improves farm productivity and helps sustainable agriculture by decreasing operational expenses while protecting the environment (Chen et al., 2025). Traditional statistical models often fall short in capturing the non-linear and uncertain relationships inherent in such systems. The review investigates modern developments in these fields while detecting research voids and emphasizing the value of the proposed approach.

### 2.1 Yield prediction for different intercropping patterns

Research on crop prediction and treatment optimization in intercropping systems has yielded significant insights (**Table 2**). The effect of canopy heterogeneity and border row proportions on light interception and light use efficiency in maize-peanut strip intercropping systems has been examined. The importance of varying row configurations, such as M2p2, M4p4, M6p6, and M8p8 is highlighted for enhancing light capture for better yield outcomes (Wang et al., 2021). Additionally, suitable solecrop and intercrop is suggested based on soil series by employing machine learning algorithms (Keerthika et al., 2024).

**Table 2:** Yield prediction across different intercropping patterns.

Contribution (citation, year)	Intercrop	Treatment (crop prediction focus)
Canopy heterogeneity with border-row proportion affects light interception and use efficiency in maize-peanut strip intercropping (Wang et al., 2021) (2021)	Maize-peanut	Border row proportion (M2p2, M4p4, M6p6, M8p8)
Crop and suitable intercrop suggestion based on soil series using machine learning algorithms (Keerthika et al., 2024) (2024)	Multiple crops	Soil classification and solecrop/intercrop prediction.

### 2.2 Traditional methods for yield prediction

With the growing global population, accurate wheat yield prediction is crucial for agricultural planning. Traditional models, like regression and mechanistic approaches, often overlook stochastic factors such as weather and technology. Here we introduced few studies based on traditional models to find research gaps, which we discussed in **Table 1**. A flexible model is developed by using random sequence analysis, achieving high accuracy (1.79%-2.75% error) without constraints on production or environmental parameters (Atamanyuk et al., 2023). Similarly mathematical linking leaf area index (LAI) and growing degree days (GDDs) have been developed to predict biomass production in arid regions, demonstrating that Cubic polynomial models performed best (Azeem and Mai, 2024). Furthermore, historical data and mathematical modeling were applied to predict crop yields in Kazakhstan, using a dynamic-statistical biomass model trained on 21 years of data (2000-2021). The model demonstrates strong correlation with official statistics 0.84 and a cross-validation correlation of 0.70, confirming its robustness against metrological variability. While these models have proven effective, their reliance on fixed mathematical relationships limits their adaptability, necessitating more flexible, data-driven approaches (Sadenova et al., 2021). The statistical models are also evaluated against CERES-Maize model for predicting maize yield under climate change in Sub-Saharan Africa (Lobell and Burke, 2010). These models excel in precipitation predictions, while panel and cross-sectional models better capture temperature effects, with accuracy improving at broader spatial scales.

### 2.3 Integration of machine learning for yield prediction in agriculture

Advancements in machine learning have significantly improved yield prediction accuracy by addressing the limitations of traditional statistical and empirical methods. Conventional techniques struggle with the nonlinearity and complexity of intercropping systems, necessitating the adoption of more adaptive and data-driven approaches (Chen et al., 2025). Machine learning optimizes crop selection and yield prediction by analyzing soil and environmental data, enhancing decision-making in farming. These models help farmers minimize losses and maximize profits through precise yield estimation (Iniyan and Jebakumar, 2022; Iniyan et al., 2023). Recent studies have explored machine learning algorithms such as RF, support Vector Regression (SVR), Gradient Boosting Machines (GBM), and Neural Networks (NN) for yield prediction demonstrating improvements in predictive performance (Jabed and Azmi Murad, 2024). In another study, machine learning algorithms such as Decision Tree, RF, XGBoost, CNN, and LSTM were applied to enhance food security through yield prediction. RF performed well, while CNN minimized the overall loss, making them the most effective models for reliable yield prediction (Sharma et al., 2023). A novel crop yield prediction framework was introduced that integrates a hybrid feature selection approach with an optimized SVR model. By employing K-means clustering, the FMIG-RFE feature selection method, and an improved Crayfish Optimization Algorithm (ICOA) for SVR hyper parameters tuning, the model achieved superior accuracy and efficiency, outperforming state-of-the art methods (Abdel-salam et al., 2024). Furthermore, performance records from Uniform Soybean Tests (UST) were used to develop an LSTM-based model incorporating pedigree data and weekly weather parameters for genotype response prediction. This model outperformed SVR-RBF, LASSO, and the USDA yield prediction model. Additionally, a temporal attention mechanism enhanced interpretability, offering valuable insights for plant breeders.

### 2.4 Soft computing approaches for yield prediction

The combination of machine learning and soft computing techniques significantly improves the predictive power of yield estimation models by integrating interpretability, adaptability, and dynamic optimization. The data-driven AI algorithms are utilized for maize yield prediction in maize-legume intercropping systems, utilizing soft computing techniques with machine learning algorithm such as symbolic regression and fuzzy logic are implemented with genetic algorithms, which resulted in higher accuracy (Agboka et al., 2022) (2022). An ANFIS-MOGA approach has been applied to optimize agricultural sustainability by simultaneously improving energy efficiency, economic returns, and environmental impact in canola to evaluate land suitability for wheat cultivation in northwestern Iran. This method identified 53.79% of land as highly suitable, with slope, soil depth, and salinity as key limiting factors (Seyedmohammadi and Navidi, 2022). Fuzzy systems offer interpretability and robustness, which are essential for complex intercropping scenarios where multiple environmental and agronomic factors interact (Val et al., 2025). Yield prediction has also been explored from an energy perspective, focusing on fault detection, systems commissioning, and efficiency evaluation in agricultural production. Studies have examined ANN and ANFIA for modeling energy use and predicting output energy, detailing data collection, energy analysis, and model design (Nabavi-Pelesaraei et al., 2021).

### 2.5 Optimization techniques for yield prediction

Optimizing techniques play a crucial role in enhancing crop yield prediction accuracy by mitigating underfitting and overfitting issues. An Improved Optimizer Function (IOF) integrated with LSTM improves model performance by refining weight adjustments and convergence. Compared to eight standard models IOF-LSTM achieves lower RMSE and MAE, outperforming CNN, RNN, and standard LSTM in crop yield prediction (Bhimavarapu et al., 2023). An intelligent agricultural optimization system integrating deep learning and hybrid optimization techniques is used to enhance crop yield prediction and pest detection. By combining GA and Particle Swarm Optimization with CNNs, RNNs, LSTMs, and GANs, the proposed method achieves superior accuracy, reducing MSE in yield prediction and improving pest detection accuracy from R^2^ 0.93 to 97.5%, outperforming conventional models in efficiency and scalability (Upreti et al., 2024). Machine learning with IoT sensor data is integrated to optimize crop production and decision making, achieving 99.50% accuracy with Bayes Net. The findings enhance yield prediction, disease detection, and cost efficiency, promoting sustainable agriculture (Elbasi et al., 2023).

### 2.6 Research GAP

Despite advancements to yield prediction for intercropping systems, there are several limitations still persisting (see **Table 3**). Traditional statistical and process based models struggle to capture the non-linear and dynamic interactions between crops, soil conditions, and climate variability. Existing machine learning models improve prediction accuracy but lack interpretability, making it difficult for farmers to understand influential factors. Additionally, most studies do not integrate optimization techniques to fine tune model performance and overcome the issues like overfitting or underfitting. There is also limited research on hybrid approaches that combine fuzzy inference systems with machine learning to handle uncertainties in intercropping systems. This study addresses gaps by proposing FOHEM that enhances prediction accuracy, incorporates interpretability methods (LIME and SHAP), and optimizes model performance using GA.

**Table 3:** Research gaps and FOHEM contributions.

Research Gap	Existing Limitation	FOHEM’s Solution
Uncertainty in yield prediction	Machine learning models lack soft computing for handling variability.	Integrates FIS with ensemble learning for better uncertainty management and robust predictions.
Limited adaptability to intercropping treatments	Yield models do not consider SS, SM, 2M2S and 2M3S variations.	FOHEM incorporates for treatment-specific predictions, optimizing yield estimates across different cropping densities.
Lack of interpretability	Machine learning models are black box without explainability.	Implements LIME & SHAP to enhance interpretability and provide transparent decision-making support.
Inefficient optimization	Standard models face overfitting and underfitting issues.	Uses GA to optimize model weights, fine tune FIS rules, and enhance model generalization.

## 3 Material and methods

This study proposes a soft computing framework that integrates fuzzy logic with machine learning for yield prediction in agriculture. The framework employs fuzzy rule generation for handling uncertainty, followed by a stacked ensemble machine learning approach for enhanced predictive accuracy. GA is utilized for optimizing the dynamic weights assignment of the components of proposed model FOHEM.

### 3.1 Description of experimental area and dataset

The dataset is acquired from the Department of National Research Center of Intercropping (NRCI) at The Islamia University of Bahawalpur, Pakistan (https://nrci.iub.edu.pk) (Raza et al., 2021; Raza et al., 2022). It is based on historical yield records for maize-soybean intercropping for both solecrop and intercrop scenarios, collected from 2018 to 2020. This experimental initiative was conducted at research farms located in Tehsil Khairpur Tame Wali and District Bahawalpur, South Punjab, Pakistan. Dataset is based on four treatments: SS, SM, 2M2S and 2M3S as shown in **Table 4** (Raza et al., 2019), based on 28 parameters and 225 records. Soil analysis was performed in laboratory, while weather data was collected using a weather station to ensure accurate environmental monitoring. For further methodological details, referenced studies provide a comprehensive description.

**Table 4:** Nomenclature.

Variable Name	Description
M_residue_biomass	maize residue biomass
bulk_density	bulk density
LAI	Leaf area index
total_biomass	Total biomass
SM	Sole maize
SS	Sole soybean
2M2S	Two rows of maize alternating with two rows of soybean
2M3S	Two rows of maize and three rows of soybean

#### 3.1.1 Model structure

**Figure 3:**
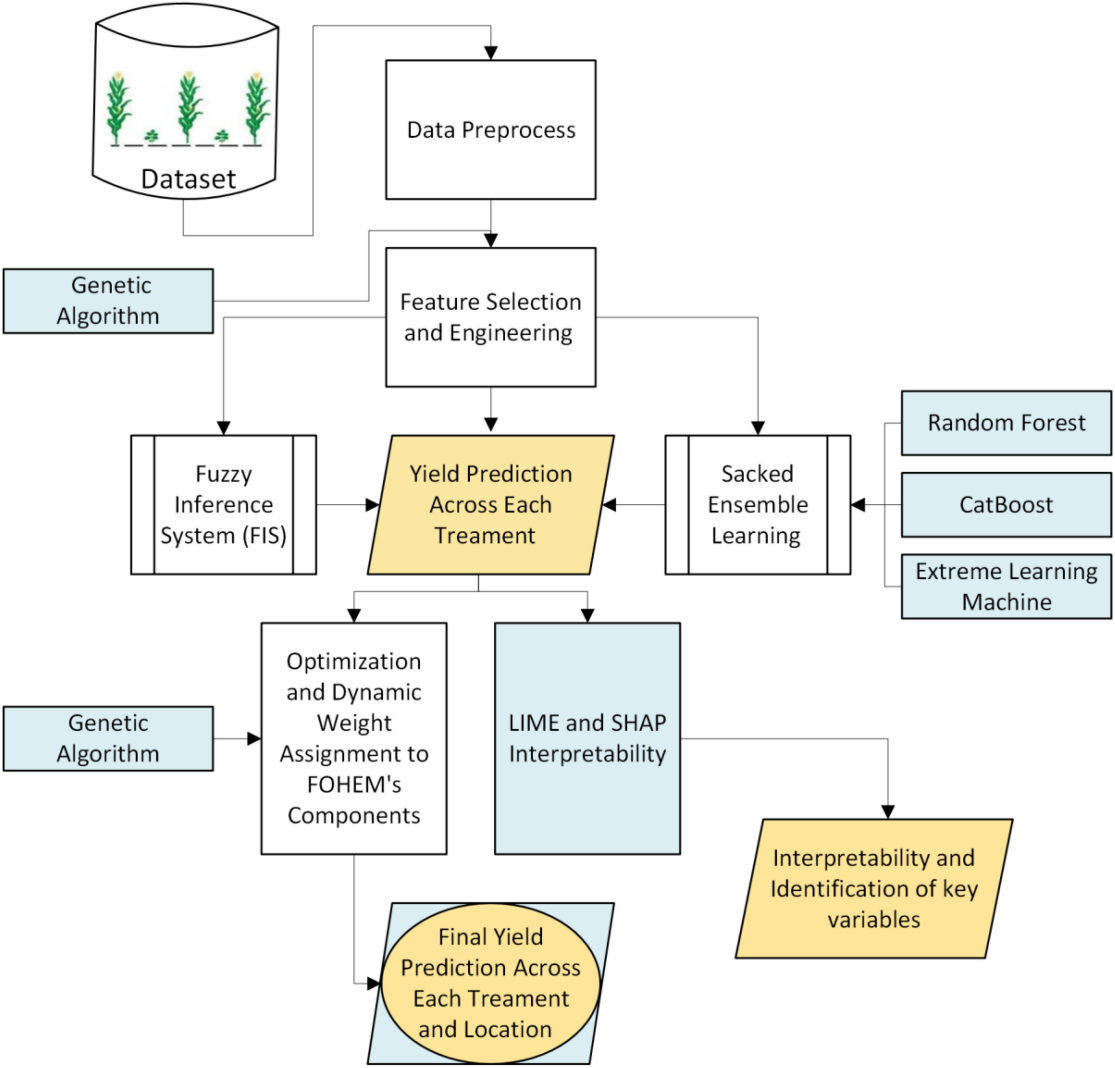
Framework of proposed model.

The proposed yield prediction model (see **Figure 3**) is structured as follows:

*Input Layer:*

The model uses several agricultural features by considering expert knowledge and previous studies based on yield prediction (Stratton et al., 2022). We categorized the parameters into three groups:

- Soil parameters: Soil organic carbon, soil percentage nitrogen, soil percent carbon, soil carbon to nitrogen ratio, bulk density, manganese, phosphorus, potassium and soil pH etc.
- Crop related variables: Total biomass, transpiration, crop nitrogen uptake, yield per plant and crop biomass etc.
- Management parameters: Treatment type, nutrient management practices, pest management practices and residue biomass etc.

The dataset was synthetically generated using expert-defined parameter ranges, ensuring controlled variability and realistic data representation.

*GA-optimized Features Selection and Engineering*

A GA-based approach is used to enhance the efficiency of feature selection and transformation (da S. Bohrer and Dorn, 2024). Traditional feature selection techniques, such as correlation analysis and RF features importance provide initial insights into relevant variables. To overcome these limitations, GA was employed for both feature selection and feature engineering, ensuring an optimized feature set that maximized predictive accuracy while minimizing overfitting risks.

In the GA-based feature selection process, the algorithm was designed to identify the most relevant subset of features while minimizing redundancy and preserving or improving predictive accuracy. Each candidate solution was encoded as a binary chromosome where a “1” denoted feature inclusion and a “0” indicated exclusion. The fitness of each chromosome was evaluated based on the model’s predictive performance. Beyond feature selection, GA was also applied feature engineering to explore optimal transformations and interactions among the selected variables. The engineered features were generated through mathematical operations such as polynomial expansions, logarithmic scaling, and interaction term construction. The fitness function ensured that only feature transformations that positively contributed to model performance were retained.

The GA was implemented with the following key parameters for both features selection and feature engineering:

- Population size: 100
- Crossover rate: 0.8
- Mutation rate: 0.02
- Selection strategy: Tournament selection
- Stopping criteria: Convergence of fitness values after 50 generations.

In each iteration, tournament selection was used to select the best-performing feature subsets or transformations. Selected solutions underwent crossover operations (rate: 0.8) to exchange genetic material, ensuring diversity in potential solutions. Mutation (rate: 0.02) was applied to introduce small random changes, allowing for exploration of new feature subsets or transformations (Hassanat et al., 2019).

The optimization process continued until the fitness values converged after 50 generations, ensuring that the best-performing features and transformations were selected. This automated approach reduced the need for manual feature selection and engineering, leading to a more efficient and robust predictive model.

The parameters settings were determined based on both prior literature and empirical experimentation.

Population Size: A population size of 100 strikes a balance between maintaining genetic diversity and ensuring computational efficiency. Recent studies have demonstrated that such size is effective in exploring the solution space without incurring excessive computational costs (Yuan et al., 2018; Haupt, 2000).

Crossover Rate: A crossover rate of 0.8 facilitates effective recombination of genetic material promoting exploration of new solutions while preserving high-quality traits from parent solutions. This rate has been recommended in recent literature for its efficacy in diverse optimization problems (Hassanat et al., 2019).

Mutation Rate: Setting the mutation rate at 0.02 introduces necessary variability into the population helping the algorithm a void local optima without disrupting convergence. Studies have found this rate to be effective in maintaining a balance between exploration and exploitation (Hassanat et al., 2019).

Selection Strategy and Stopping Criteria: Tournament selection is known for maintaining diversity and preventing premature convergence. The stopping criterion of 50 generations was determined based on preliminary experiments, which showed that fitness values typically stabilized within this range, indicating convergence.

These parameters choices were further validated through preliminary testing on our dataset, confirming their suitability in achieving robust and efficient feature selection and engineering.

*Fuzzy Rule Generation Layer*

Fuzzy rules are generated based on expert knowledge and features selection by GA. The features selected by GA were chosen as inputs for membership function design because they demonstrated the highest impact on yield variability. By optimizing feature selection with a yield prediction objective, GA ensures that the fuzzy rules built from these variables are quietly relevant and enhance prediction reliability (Aditya Shastry and Sanjay, 2021; Altarabichi et al., 2023). The aim is to represent the qualitative aspects of agriculture that can influence yield. Each fuzzy rule can be expressed mathematically (Equations 1-5) as follows:


$$\begin{array}{c} {\text{If X}}_{1}\;{\text{is A}}_{1}\;{\text{AND X}}_{2}\;{\text{is A}}_{2}\\ \text{Then Y is B} \end{array}$$

where,

- X_1_, X_2_ are input features (selected features)
- A_1_, A_2_ are the fuzzy sets corresponding to these features.
- Y is predicted yield.
- B is the fuzzy set of the yield.

This can be formally represented as:


(1) $$Y=B\;if\;\text{min}(\mu_{A1}(X_{1})\times \mu_{A2}(X_{2}))$$

where,

-
$\left(\mu_{A1}(X_{1}\right)$ and $\mu_{A2}(X_{2})$ are the membership functions of the fuzzy sets A_1_ and A_2_, respectively, for the input X_1_ and X_2_.
- The main function is used in fuzzy logic to combine multiple fuzzy sets.

The fuzzy sets A_i_ (i=1, 2…) can be defined using membership functions. For example, a triangular membership function can be defined as:


(2) $$\mu_{A\;}(X)=\{\begin{array}{cc} 0,\; & if\;x<0\\ \frac{x-a}{b-a}-,\; & if\;a\leq x<b\\ \frac{c-x}{c-b}-,\; & if\;b\leq x\leq c\\ 0, & if\;x\geq c \end{array},$$

where,

- a, b, c are the parameters that define the triangular shape of the membership function.
- The function value ranges from 0 to 1 as a, b, and c.

*Yield Prediction Layer*

In machine learning yield prediction layer evaluates predictions from ensemble learning models, specifically RF, CatBoost and ELM. These models process the original input features independently to generate yield predictions (Hussain et al., 2024; Ikram et al., 2025). For optimal output, the ensemble outputs are compared with predictions from the FIS. A GA dynamically optimizes weights based on performance metrics, combining the FIS and ensemble predictions. The final model output is calculated as:


(3) $$\hat{\text{Y}}=\sum_{i=0}^{N}{\text{w}}_{i}\times ({\text{f}}_{i}(X))$$

where,

- N is the number of components of FOHEM, including the FIS and the stacked ensemble learners.
- W_i_ are the weights assigned to each component of the FOHEM.
- F_i_ (X) is the prediction from ith component for input features X.

GA is applied to dynamically optimize the weights of the components of FOHEM (FIS and stacked ensemble learning), ensuring the best possible yield prediction. The optimization process can be described as follows:

- Initialization: A population of candidate solutions is generated, where each individual represents a set of weight assigned to the components of FOHEM.
- Fitness function: The optimization process can be described as follows:

(4) $$Fitness=\sum_{i=0}^{N}\hat{\text{Y}}(treatment_{i},location_{i})$$

where, N is the total number of treatments and Ŷ is predicted yield.

- *Selection, Crossover, Mutation*: The best performing solutions (weight sets) are selected for reproduction. These solutions undergo crossover and mutation to explore new solutions.
- *Termination:* Iterations continue until the convergence criterion is met, which is typically the achievement of the best fitness (optimal weight set for FOHEM components).

*Final Weighted Output*

Once the GA has completed its optimization process, it returns the optimal weights w_1_ and w_2_. These weights are then applied to the FIS and ensemble outputs to compute the final combined prediction.


(5) $${\hat{\text{Y}}}_{\text{Final}}=\;({\text{w}}_{1}\times {\text{Y}}_{\text{FIS}})+({\text{w}}_{2}\times {\text{Y}}_{\text{Ensemble}})$$

This integrated output ${\hat{\text{Y}}}_{Final}$ is the final prediction for crop yield, which benefits from the strengths of both the fuzzy based approach and machine learning based approach.

#### 3.1.2 Algorithm of proposed model

The proposed model follows this structured algorithm as shown in **Algorithm 1**:

**Algorithm 1:** FOHEM Algorithm for Yield Prediction.

No of steps	Process	Details
1	Input data acquisition	-Collect datasets from NRCI department. -Features selection and engineering performed by GA.
2	Fuzzy rule generation	-Define fuzzy sets for selected features based on expert knowledge and dataset analysis. -Create fuzzy rules using (2).
3	Stacked ensemble model training	-Train multiple base learners (RF, CatBoost, ELM) using selected features. -Generate predictions from each learner.
4	Integration of predictions	-Combine outputs from FIS and stacked ensemble models using a weighted average. -Use GA to improve model performance and dynamically assigning weights to FIS and stacked ensemble machine learning according to their performances in FOHEM prediction.
5	Optimization	-Initialize a population of candidate solution, where each individual represents a set of weights for the components of FOHEM. -Define a fitness function to evaluate the performance of each solution. -Apply GA to dynamically optimize the weights of the components of FOHEM, ensuring improved yield prediction accuracy. -Terminate when convergence is reached.
6	Prediction	Once the optimal weights identified, predictions are made for the test data, combining the outputs from the two models.
7	Model interpretability	Use LIME and SHAP for interpretability: -SHAP for global insights into feature importance. -LIME for local explanations of parameter predictions.
8	Final yield prediction	The final yield predictions are produced, with performance evaluation and insights from SHAP and LIME to understand the model’s decision making process.

## 4 Results section

The results of this study are presented in a structured manner, starting with a treatment-wise comparison of yield predictions for both locations. This is followed by an evaluation of the performance of the FIS and stacked ensemble learning model. Afterward, the optimization of dynamic weights for FOHEM using GA is discussed. The interpretability of the model is further enhanced through the use of LIME and SHAP, allowing for a better understanding of the influential parameters. Finally, the integrated model’s overall performance is validated by comparing existing models.

**Figure 4:**
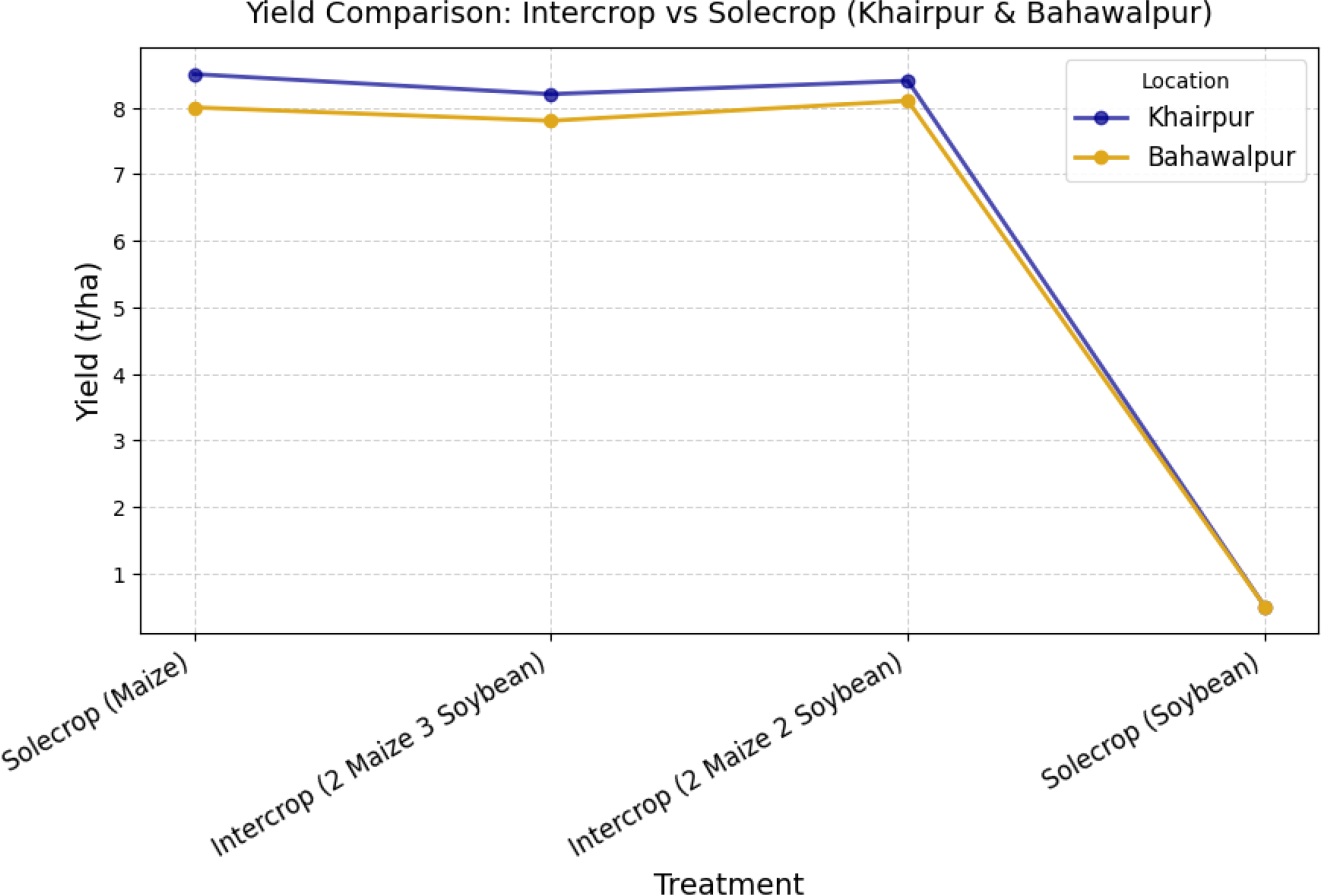
Treatment wise average yield (tons/ha) comparison across each location.

The yield performance analysis in **Figure 4** highlights the advantages of intercropping over sole cropping, particularly in resource utilization and stability across different locations. The maize-dominant treatment (SM) exhibited higher yields compared to soybean alone (SS) reaffirming maize’s resilience and adaptability to local conditions. However, intercropping treatments 2M2S and 2M3S demonstrated competitive productivity, with yields approaching those of sole maize while offering diversification benefits. A trend observed is the location based variation in yields, where Khairpur consistently outperformed Bahawalpur across all treatments. This suggests site-specific influences such as soil properties, microclimate variations, and management practices. Additionally, the yield stability in intercropped treatments indicates a potential buffering effect against environmental stress, reinforcing intercropping’s role in sustainable yield optimization.

**Figure 5:**
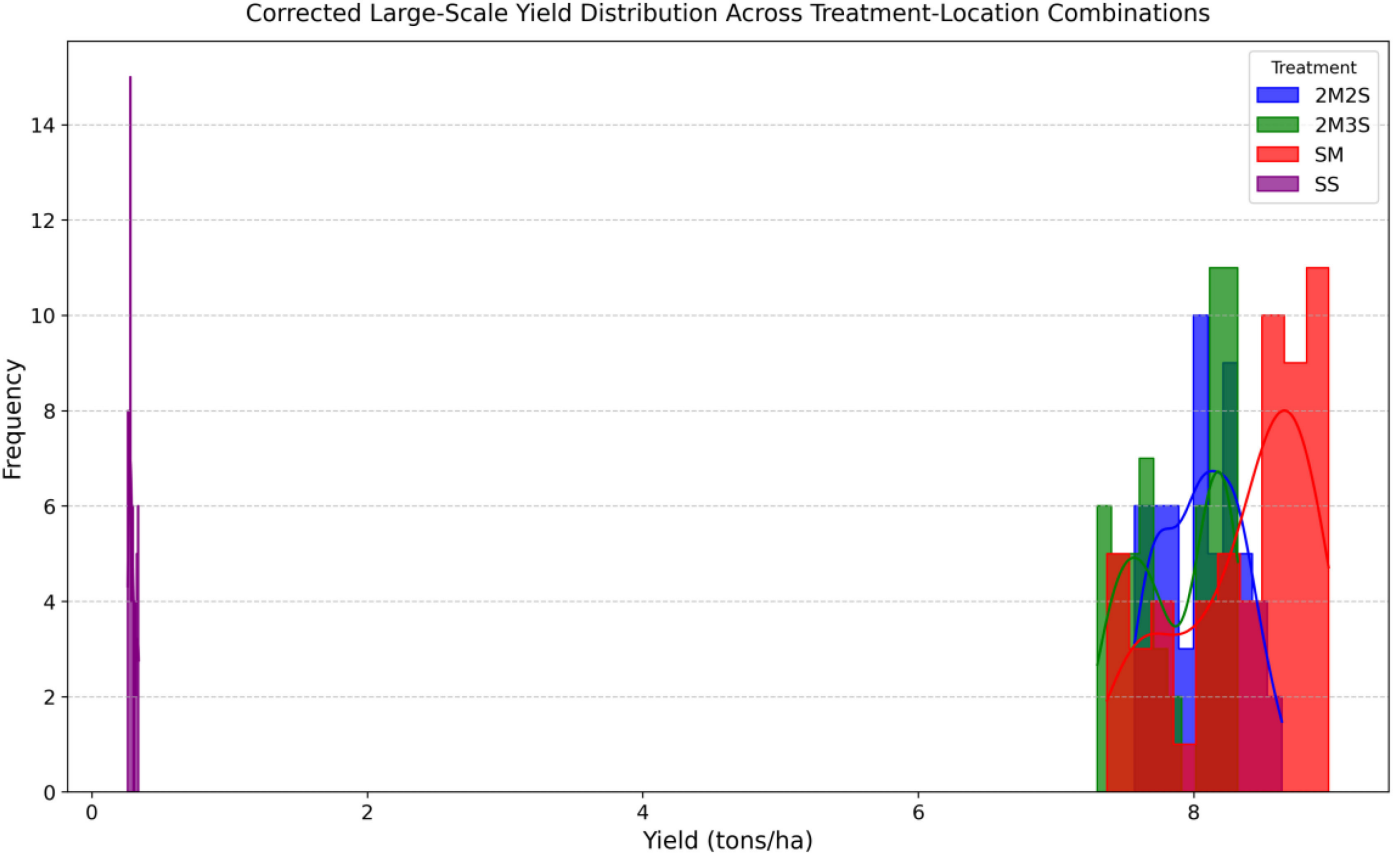
Yield distribution across each treatment.

Overall intercropping outperformed sole cropping, particularly for soybean, suggesting better resource utilization and complementary growth patterns. The histogram in **Figure 5** reveals distinct yield variations across different treatment-location combinations. The 2M2S treatment demonstrating the highest yield stability across locations. This suggests that structured row arrangements contribute to better nutrient distribution and efficient space utilization. The distribution patterns across treatments indicate that yield variability is strongly influenced by planting strategies. The relatively lower yield of SS emphasizes its sensitivity to environmental and soil constrains, while intercropping appears to mitigate these limitations. Furthermore, location-specific factors contribute to yield differences, underscoring the need for tailored agronomic management practices to maximize productivity.

**Figure 6:**
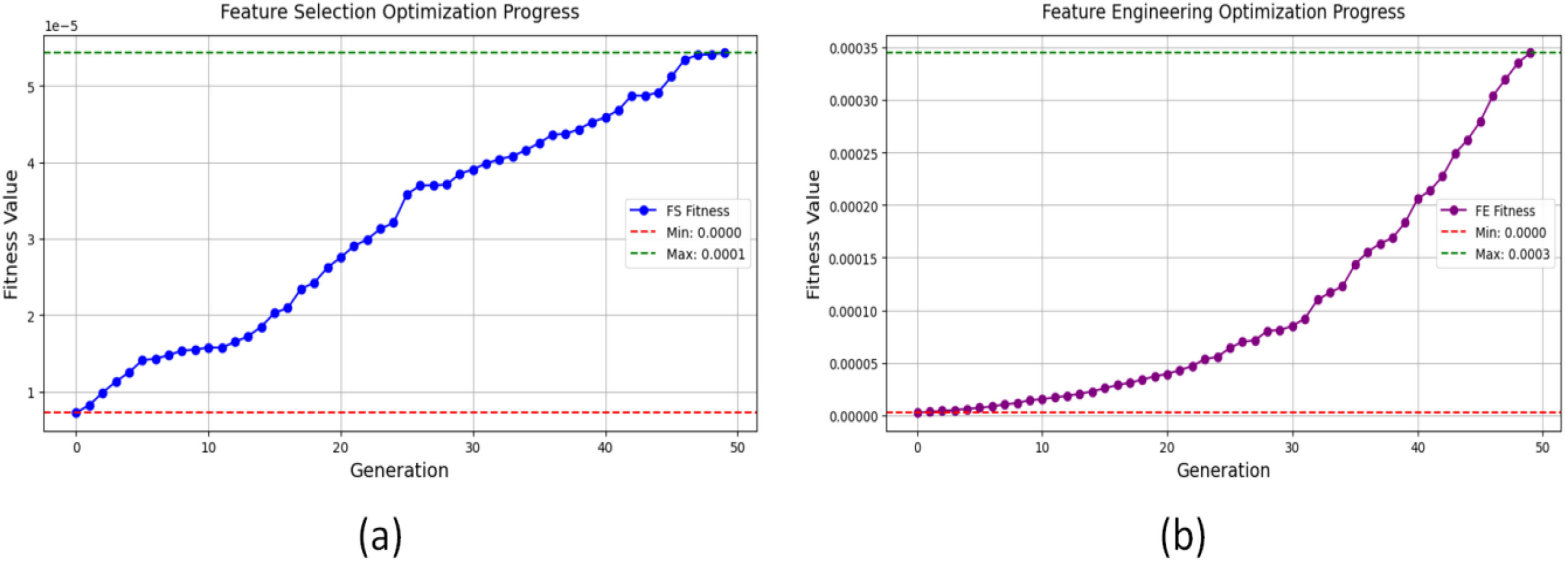
Genetic Algorithm (GA)-based optimization: **(a)** feature selection progress, and **(b)** feature engineering progress.

Unlike RF-based feature ranking, which prioritize features based on individual performance, GA optimizes feature selection holistically, ensuring that the chosen variables contribute maximally to yield prediction accuracy. The GA-based feature selection and engineering process demonstrated significant improvements in predictive performance and feature efficiency. The feature selection optimization progress is given in **Figure 6a**, showed that the initial fitness values remained relatively low during early iterations. However, as the algorithm evolved, the selected features subsets demonstrated steady improvements in model performance, ultimately reaching a final optimized fitness. This increase in predictive accuracy confirmed that the GA-selected features provided better model generalization compared to traditional feature selection techniques.

Similarly, the feature engineering optimization, presented in **Figure 6b**, highlighted how GA systematically identified the most important transformations and interactions. The fitness values exhibited a progressive upward trend, indicating that the engineered features significantly contributed to error reduction and improvement in R^2^. The GA-engineered features outperformed traditional methods in predicting errors while improving model robustness thus enhancing the model’s reliability for yield prediction. These approaches ensured that the selected features were not only statistically relevant but also functionally meaningful in the context of yield prediction. The optimization process produced a stronger and more efficient solution for modeling agricultural intercropping systems that also provided scalability. The GA-selected features formed the basis for developing the membership functions because they demonstrated the highest impact on yield results.

For example total_biomass, together with M_residue_biomass and transpiration proved to be essential yield-determining factors, which were included in the fuzzy systems. These membership functions are designed to capture their non-linear effects on yield prediction. The FOHEM model combines FIS with ensemble learning to effectively manage yield prediction uncertainties through stacked ensemble learning. The fuzzy logic component accurately modeled the uncertainties, and improved prediction reliability, specifically for treatment with high variability.

**Figure 7:**
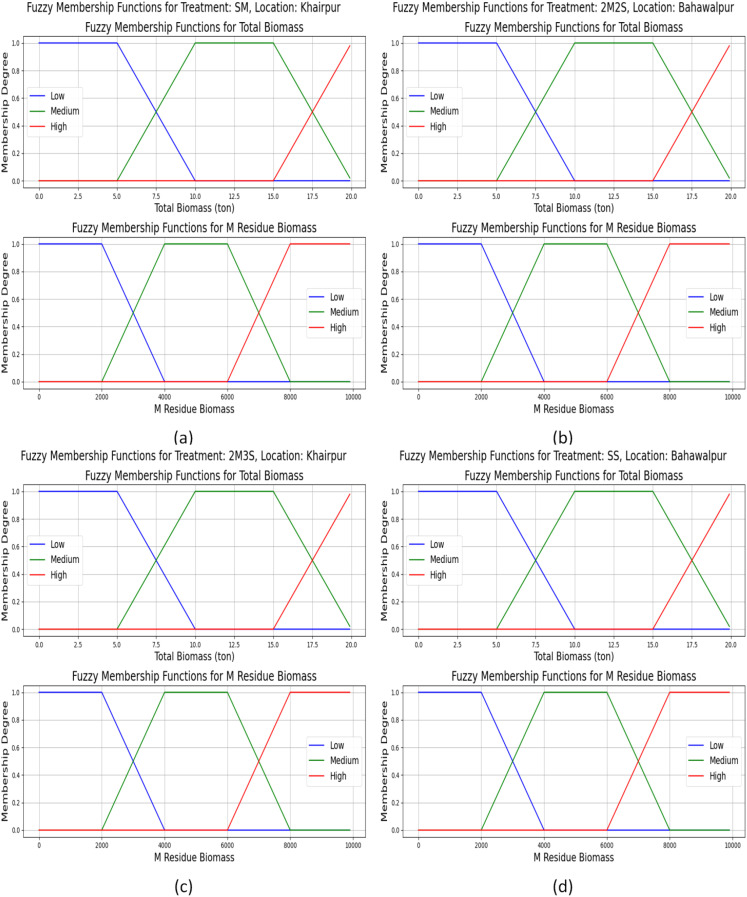
Membership functions for total_biomass and M_residue_biomass across treatments and locations: **(a)** SM at Khairpur, **(b)** 2M2S at Bahawalpur, **(c)** 2M3S at Khairpur, and **(d)** SS at Bahawalpur. These graphs highlight the variations in total_biomass and M_residue_biomass under different conditions showing how Khairpur treatments exhibit higher biomass and residue levels compared to Bahawalpur.

The few fuzzy membership functions of M_residue_biomass and total_biomass for various treatments (SM, SS, 2M2S and 2M3S) across two locations (Khairpur and Bahawalpur) are presented in **Figure 7**. The membership functions for total biomass indicate three categories: low (0–10 tons), medium (5–20 tons), and high (15–20 tons). The 2M2S and 2M3S treatments in Khairpur show higher biomass values, while Bahawalpur, especially under the SS treatment, exhibits lower biomass levels. Similarly, the M_residue_biomass graphs show categories ranging from low (0–4000 kg/ha) to high (6000–10000 kg/ha). Khairpur consistently shows higher residue_biomass, particularly in the SM and 2M3S treatments, whereas Bahawalpur shows lower residue values, with the SS treatment showing no residue biomass. These graphs highlight the impact of treatment and location on biomass production and residue levels, offering insights for optimizing intercropping systems. Hence, the fuzzy classification approach effectively captures uncertainty and variability in biomass data, offering a more adaptable framework for analyzing intercropping systems. This method enhances decision-making by providing a gradual and interpretable categorization of biomass levels rather than relying on rigid, predefined thresholds.

**Figure 8:**
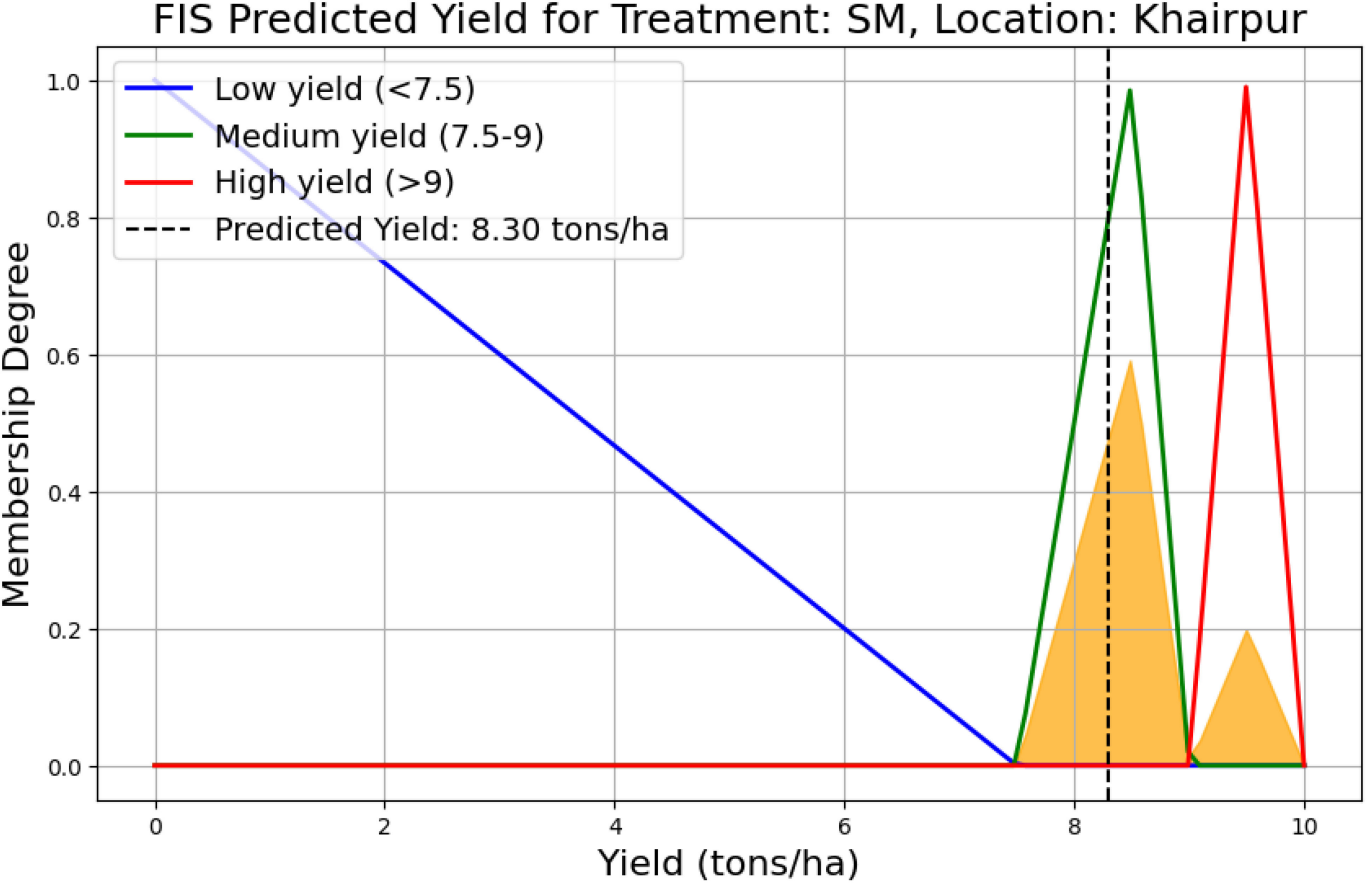
Predicted yield for SM treatment in Khairpur based on the FIS, with low, medium, and high yield categories defined. The predicted yield of 8.3 tons/ha falls within the medium yield range (7.5 to 9 tons/ha).

The graph in **Figure 8** illustrates the predicted yield for the SM treatment in Khairpur using a FIS. The membership functions low, medium, and high yield are defined based on the yield range, low yield for values below 7.5 tons/ha, medium yield for values between 7.5 and 9 tons/ha, and high yield for values above 9 tons/ha up to 10 tons/ha. The predicted yield of 8.3 tons/ha falling within the medium yield range is highlighted by a vertical dashed line. The shaded area under the curve indicates the aggregation of fuzzy rule outputs, showing the degree of membership across different yield categories. This graph provides a clear visualization of the yield prediction for this specific treatment and location.

The top three best performing algorithms were selected as the base models in the stacked ensemble for FOHEM (see **Table 5**). These models were chosen based on their ability to minimize errors while maintaining strong predictive performance.

**Table 5:** Model selection for stacked ensemble learning for FOHEM.

Model	MSE	MAE	R²
RF	0.03625	0.16963	0.99575
CatBoost	0.05016	0.20364	0.99354
ELM	0.16212	0.21954	0.98650
KNN	0.17838	0.29829	0.98562
MLR	0.10319	0.26133	0.98185
MLP Regressor	0.87895	0.75830	0.92692
SVR	1.08413	0.76281	0.90986
TabNet	50.8463	6.25442	-3.23051
Neural Network	345.59571	14.83937	-26.84757

RF was selected for its outstanding performance in terms of MSE (0.0563) and R^2^ (0.9958). As an ensemble method it uses multiple decision trees which are highly effective for capturing complex nonlinear relationships and avoiding overfitting. Its ability to model high dimensional and varied structure data makes it an ideal base model for this task. The low MSE and MAE indicate that RF produces highly accurate predictions with minimal residual error.

CatBoost is another strong performer showing MSE (0.0802) and R^2^ (0.9935). It is particularly effective with categorical data and use gradient boosting framework, which is effective in capturing hidden patterns and interactions in the data. CatBoost’s robustness to noisy data and its relatively low MAE, highlight its ability to maintain consistent prediction accuracy across varying data conditions. The slightly higher error values compared to RF suggest that CatBoost may be more sensitive to data distribution changes, but it still remains a top performer.

ELM was included for its exceptional speed and ability to handle non-linear data effectively. It achieved an R^2^ of 0.98650, with an MSE of 0.16212. However, its MAE of 0.21945 indicates slightly higher average error in individual predictions compared to RF and CatBoost. Unlike traditional neural networks, ELM employs single layer feedforward architecture with randomly assigned weights, allowing for rapid training while maintaining high accuracy. The relatively higher MSE suggests that while ELM is effective in generalization, it may struggle slightly with capturing finer variations in the data.

**Figure 9:**
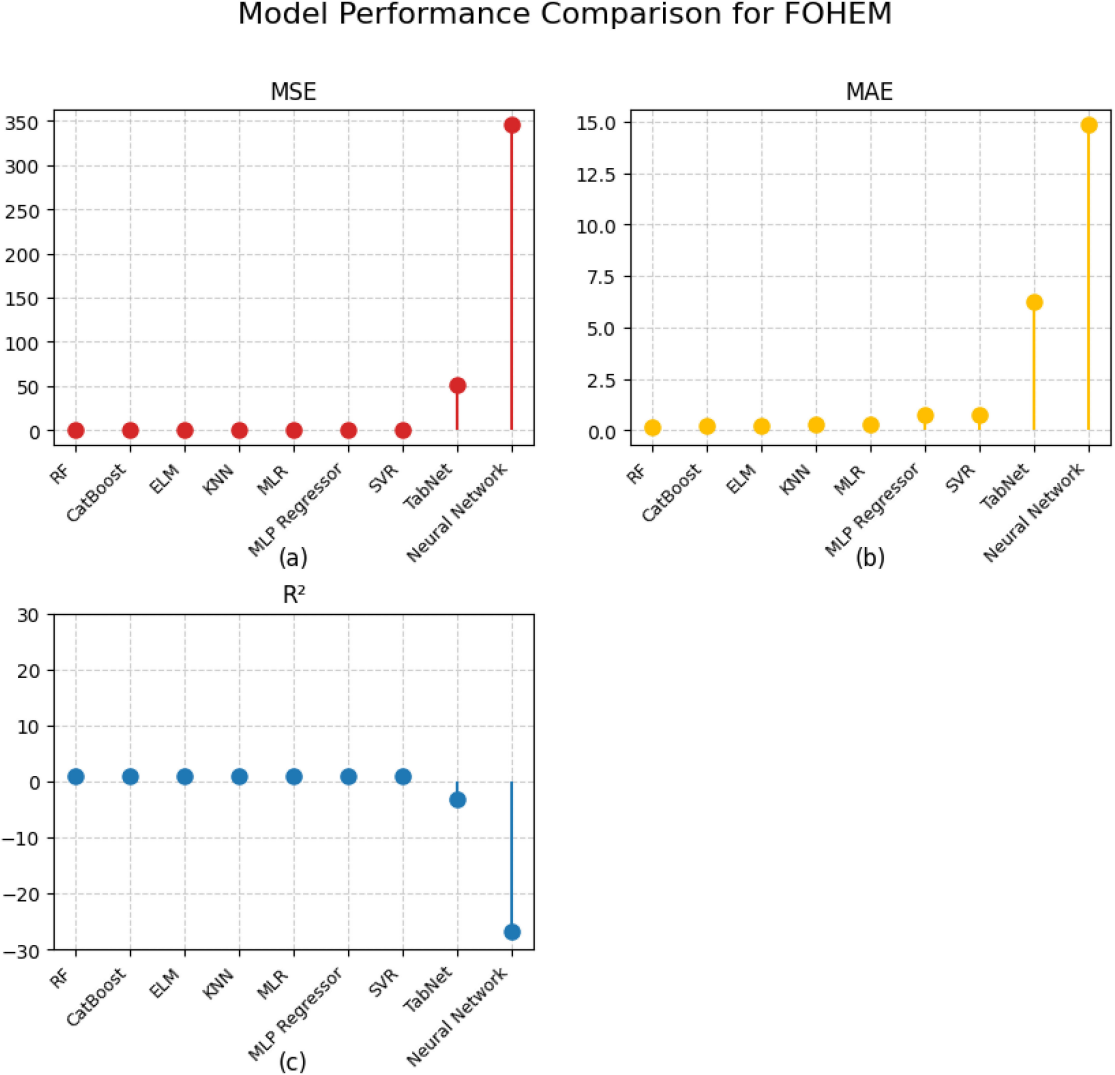
Performance comparison of various algorithms: **(a)** MSE, **(b)** MAE and **(c)** R^2^.

These results are also represented in **Figure 9**.

A comparative analysis of other models further highlights the importance of selecting base learners with the lowest error values. For instance, KNN and MLR showed higher MSE values of 0.17838 and 0.10319, respectively, indicating that they introduced greater variance in their predictions. Similarly, MLP Regressor, SVR, and TabNet displayed significantly larger errors, with TabNet and a general neural network model producing extremely high MSE values of 50.8463 and 345.59571, respectively. These high errors demonstrate their poor generalization capabilities in this specific yield prediction task.

The selection of RF, CatBoost, and ELM as base models was thus driven by their ability to minimize both systematic and random errors, ensuring robust performance in the stacked ensemble. The relatively low MSE and MAE values across these models indicate reduced variance and bias in their predictions, making them suitable components for further optimization within FOHEM.

The results are also visually summarized in **Figure 9**, which clearly shows the superior performance of RF, CatBoost, and ELM compared to other models.

**Figure 10:**
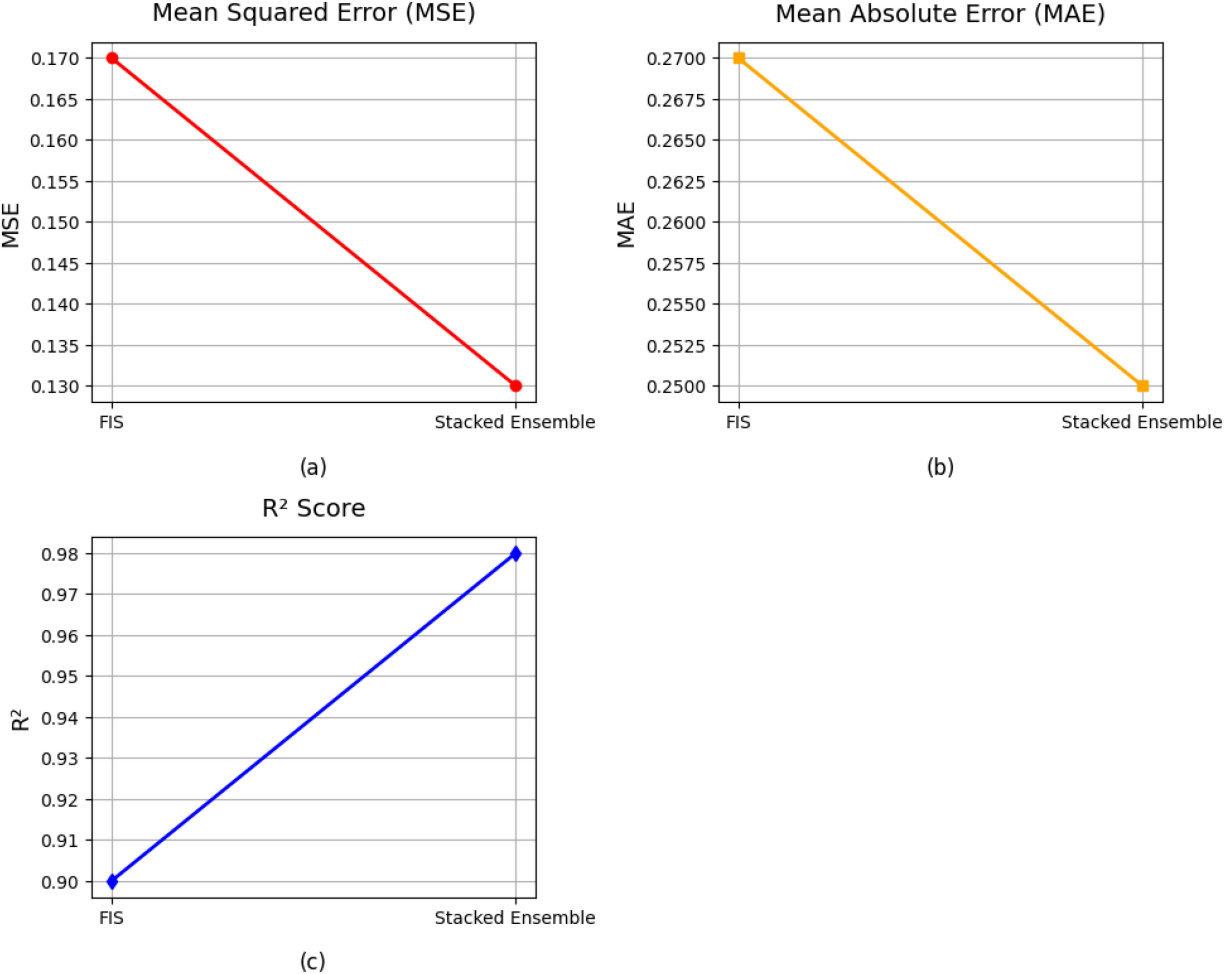
Individual performance of base learners in stacked ensemble learning with FIS and proposed model FOHEM. The graphs **(a-c)** illustrated the MSE, MAE and R^2^.

In **Figure 10**, graphs (a, b, c) compare the performance of the stacked ensemble model and the FIS using three metrics: MSE, MAE and R^2^. The stacked ensemble model consistently outperforms the FIS across all evaluation metrics, demonstrating lower error values (MSE and MAE) and a higher R^2^ of 0.9893. This superior performance highlights the effectiveness of combining multiple base learners to enhance prediction accuracy and model generalization. The integration of fuzzy logic with ensemble learning provides a hybrid approach that uses the strengths of methodologies, improving model robustness and reliability. While FIS contributes to capturing nonlinear interactions, the stacked ensemble further refines predictions by reducing residual errors. These findings underscore the advantages of hybrid modeling techniques in complex yield prediction scenarios.

**Figure 11:**
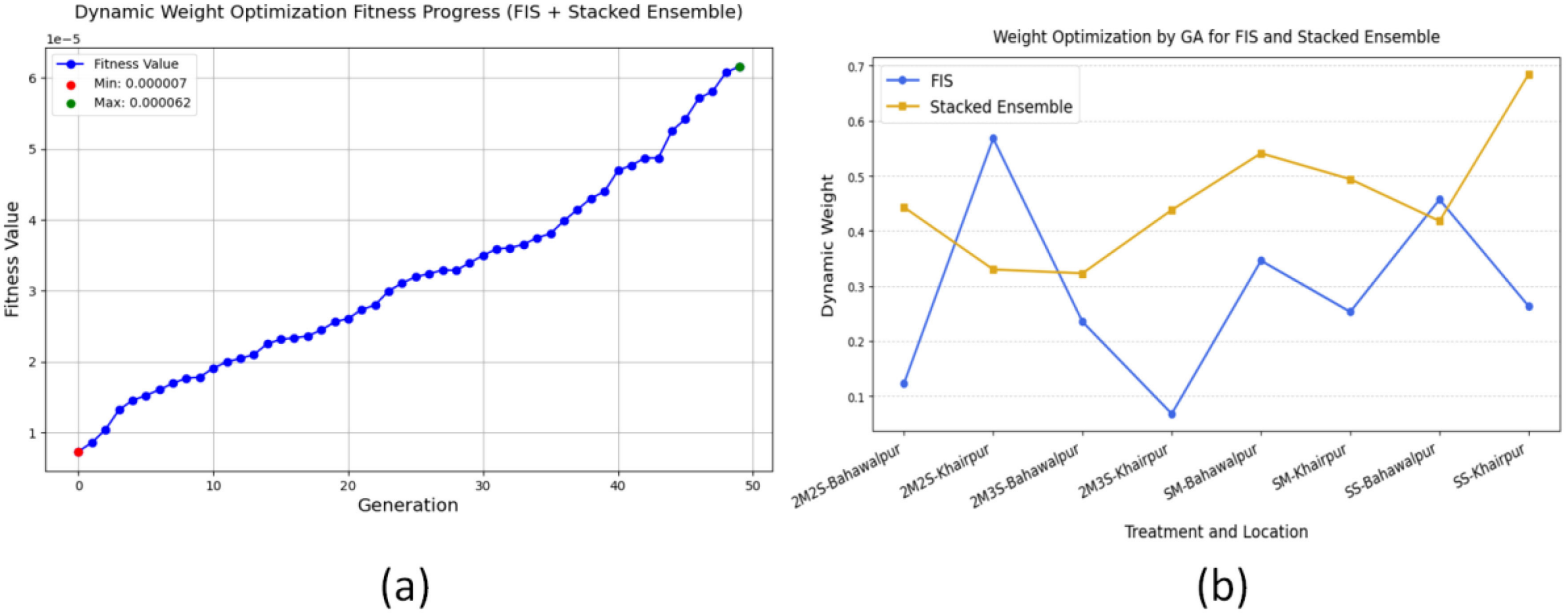
Dynamic weight optimization of the hybrid model FOHEM using GA. **(a)** Fitness graph. **(b)** Optimized weight allocation.

The **Figure 11a** illustrates the GA fitness performance for optimizing dynamic weights is an integrated system combining a FIS and stacked ensemble learning algorithms. The optimization process demonstrates enhanced total yield performance through the fitness metric across 50 generations. The optimization process starts from a low initial fitness value before GA uses its optimization capabilities to enhance the weight distribution. The 30^th^ generations marks the point where GA achieves its objective of enhancing model prediction capability as fitness growth accelerates. The highest fitness value obtained during the last generation demonstrates that dynamic weight optimization successfully enhances model reliability and accuracy. The dynamic weight distribution mechanism enables FIS and stacked ensemble to work together effectively for yield prediction by using their individual strengths. The GA-driven optimization proves its efficiency at enhancing hybrid model performance which results in a reliable predictive accuracy solution for agronomic applications.

The weight allocation process adapts its approach according to treatment-location combinations as shown in **Figure 11b**. The FIS model and stacked ensemble receive different levels of importance because each model performs best at different conditions. Treatments in Bahawalpur and Khairpur exhibit distinct allocation patterns, indicating that GA optimally balances the contributions of both models to maximize yield prediction accuracy. This adaptive approach strengthens model reliability through a mechanism which allows the most dependable predictor to determine the final yield estimates.

**Figure 12:**
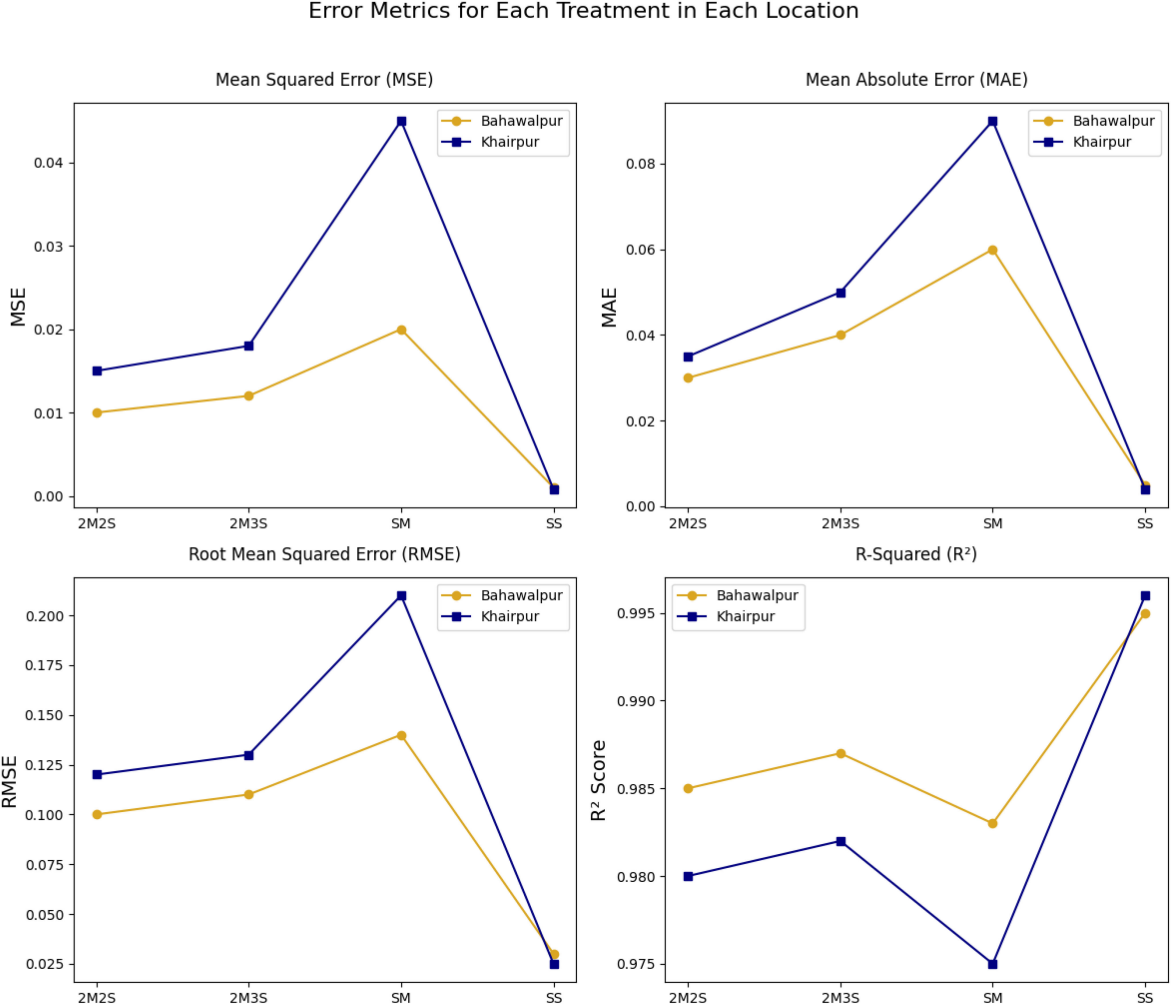
Comparison of performances of different treatments across two locations (Khairpur and Bahawalpur) using MSE, MAE, RMSE and R^2^.

The FOHEM model exhibits varying levels of prediction accuracy across different treatment-location combinations given in **Figure 12**. The SM treatment in Khairpur shows the highest error values (MSE, MAE, and RMSE), indicating greater variability in yield predictions. This suggests that factors influencing maize yield in this region contribute to higher prediction deviations. Conversely, SS treatment achieves the lowest errors, demonstrating strong model accuracy and stable yield predictions. A clear trend emerges in RMSE values, where higher errors for SM reinforce its greater prediction variance, while lower values for SS confirm its consistency. The R^2^ scores further highlight the model’s predictive reliability, with SS achieving near perfect alignment with observed data. However, the lower R^2^ for SM in Khairpur indicates areas where model refinement may be necessary. These insights underscore the importance of optimizing both treatment and location to enhance predictive precision in agricultural yield modeling.

**Table 6** showing that the model explains nearly all the variance in the yield data.

**Table 6:** Overall performance of FOHEM.

Model	MSE	MAE	RMSE	R^2^
FOHEM (Proposed model)	0.014277	0.239168	.119485	0.9892

Overall, these results confirm the effectiveness of the FOHEM model in predicting agricultural yields, providing reliable estimates across different treatments and geographic locations.

**Figure 13:**
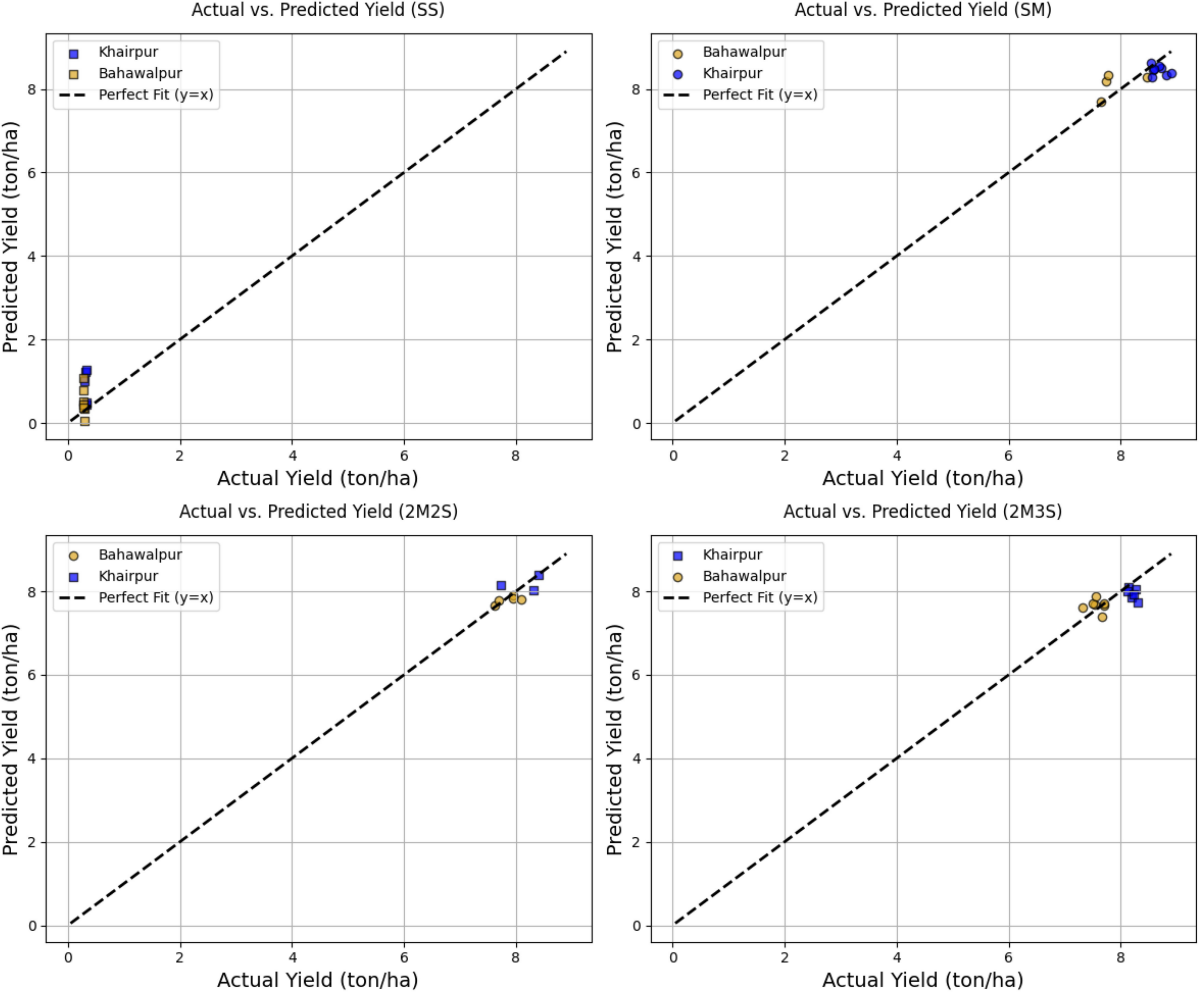
Performance evaluation of FOHEM based on actual vs. predicted yield (tons/ha) across intercropping treatments and locations (Bahawalpur and Khairpur). The top row displays results for treatments SS (left) and SM (right), while the bottom row shows 2M2S (left) and 2M3S (right).

The relationship between actual and predicted yields for different intercropping treatments across two locations is given in **Figure 13**. Each subplot represents a specific treatment SS, SM, 2M2S and 2M3S. The data points are two colors representing each location. Similarly each marker shape showing crops in each treatment. A regression line is included in each graph to indicate the ideal correlation between actual and predicted values. Most data points closely align with this line, demonstrating a high prediction accuracy of approximately 99%, indicating that the model effectively captures yield variations. However, some deviations are observed, particularly in the lower yield range, suggesting localized inconsistencies in model performance across different treatments and locations. The decision to present scatter plots separately for each treatment enhances the interpretability of results, allowing for a more detailed assessment of model performance in various intercropping scenarios.

In **Table 7**, the results of five-fold cross-validation for the proposed model with R^2^ are presented for each fold. The model demonstrates high predictive performance, with R^2^ values consistently close to 0.99 across all validation folds. The mean R^2^ score of 0.9873 indicates strong generalization ability, confirming the robustness and reliability of the model in predicting yield within the intercropping systems. These results validate the effectiveness of the integrated machine learning and FIS approach, minimizing the risk of overfitting while ensuring accurate yield estimation.

**Table 7:** Comparative analysis of FOHEM with prior model (Agboka et al., 2022).

Fold	R^2^ score
1	0.9853
2	0.9881
3	0.9865
4	0.9892
5	0.9874
Mean R^2^	0.9873

**Figure 14:**
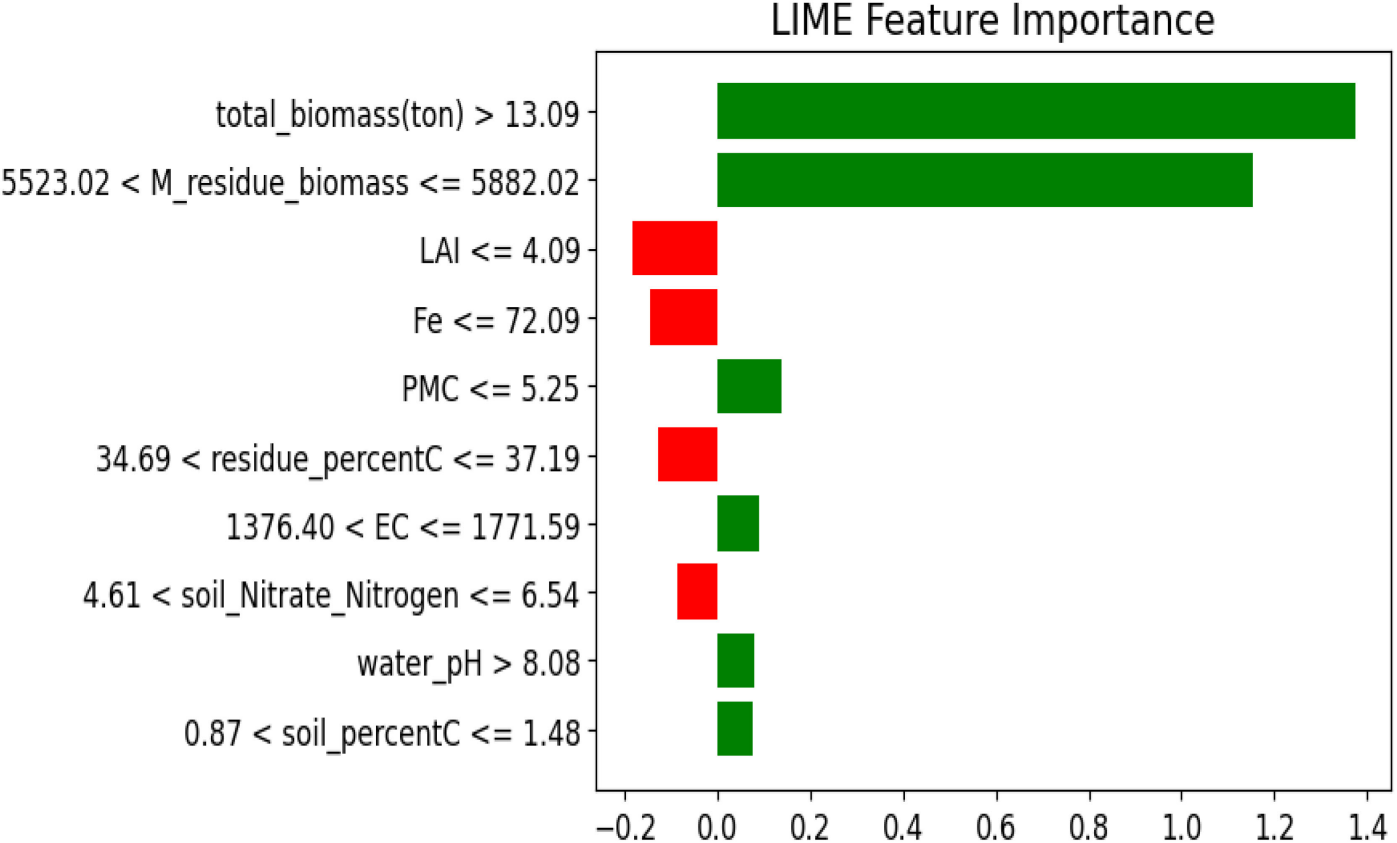
Feature importance analysis based on LIME.

**Figure 15:**
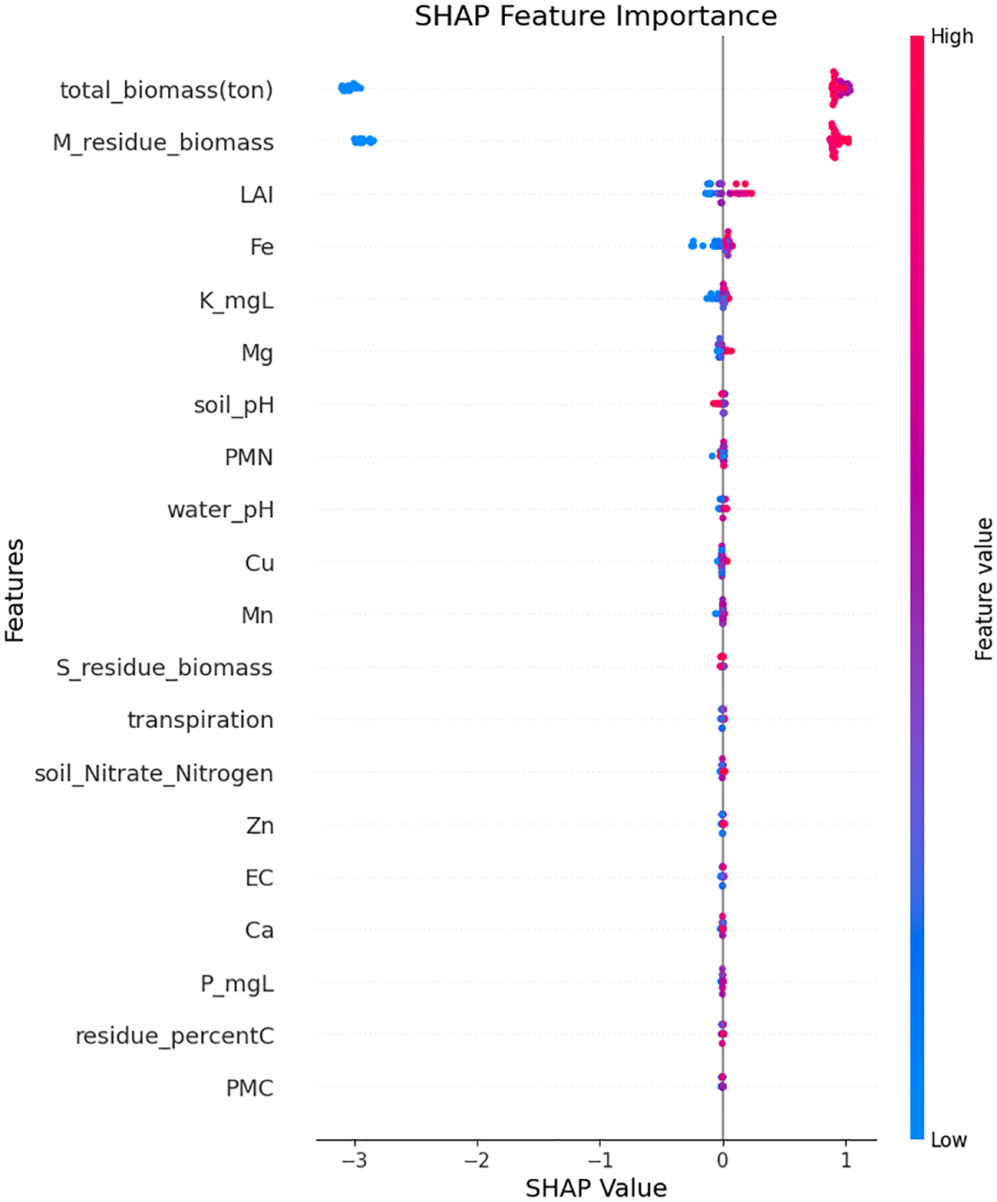
Feature importance analysis based on SHAP.

The LIME and SHAP analysis highlights influential features that have positive and negative effects on the prediction of crop yield. Such techniques give interpretability through feature importance and contribution, which gives a deeper understanding of how each of the input variables affects the result. total_biomass is the most important positive effect with values greater than 13.09 influencing the yield positively. Residue_ biomass also plays an important role in showing that management of crop residues in a right manner enhances the soil health and yield capacity. Furthermore, the features such as PMC, soil_pH and water_pH are positively influencing the prediction. On the negative side, the highest impact is associated with high iron content, which might be toxic. Similarly, a low LAI and higher clay content negatively affect yield by reducing plant growth efficiency and limiting root penetration. These findings highlight the importance of biomass and nutrient management, while monitoring soil toxicity to enhance crop yield. The graph (**Figures 14** and **15**) visually represents these influences, with positive and negative contributions, clearly illustrated.

To provide a comprehensive understanding of the difference between proposed model FOHEM and the existing model (Agboka et al., 2022) based on use of Symbolic Regression (SR) as machine learning model, Fuzzy system and GA as soft computing techniques, a detailed comparison is given in following **Table 8**.

**Table 8:** Comparative analysis of FOHEM with prior model (Agboka et al., 2022).

Aspect	FOHEM	Prior model
Model complexity	Moderate: combines FIS, GA and ensemble machine learning models.	Moderate: Uses interpretable mathematical equations, optimized by GA, to establish relationships between climatic and edaphic variables and maize yield.
Adaptability	Highly adaptable: Dynamically adjust weights to optimize performance across treatment groups.	Limited adaptability: Designed for general agro ecological systems, not specific to treatments.
Interpretability	High: Uses LIME and SHAP for feature importance and decision making.	Moderate: Relies on inherent simplicity for model interpretation.
Predictive accuracy	R^2^: 0.9892. Exceptional accuracy for diverse treatments.	R^2^ >0.9 High accuracy but focused on generalized predictions.
Scope of application	Treatment specific yield prediction	Regional maize yield prediction for generalized cropping systems like maize-legume intercropping (MLI) and push-pull technology (PPT)
Performance on treatments	Higher performance in intercropping systems, accommodating planting density and pattern variations.	Perform well for monocropping and general agro ecological systems but lacks treatment-level nuance.
Usability for decision making	High: Provides effective solutions for specific practices in intercropping systems.	Moderate: Suitable for broad policy level guidance but lacks treatment-specific insights.

## 5 Discussion

The results of this study highlighted the significant advancement in yield prediction for maize-soybean intercropping systems through the integration of innovative methodologies such as stacked ensemble learning, FIS, and GA optimization. This integration is based on the strengths of both approaches. FIS is designed to capture expert knowledge and rule-based decision making, while machine learning models excel at identifying complex patterns and nonlinear relationships in data. This hybrid framework ensures that data-driven learning is enhanced by domain-specific reasoning, leading to a more interpretable and reliable yield prediction systems. GA was chosen to optimize membership functions by selecting features that contribute most significantly to yield prediction. This approach ensures that the fuzzy system is built upon variables that only show high correlation with yield but also enhance decision making in agronomic contexts.

The findings highlight that intercropping treatment outperforms sole cropping across different locations, with treatments such as 2M2S and 2M3S shows higher productivity. This demonstrated the advantages of intercropping in optimizing resources and using complementary growth patterns between crops. The study employed advanced explainability tools, LIME and SHAP, to provide interpretability of the model and identify influential parameters such as total biomass, residue biomass and PMC, which positively impact yield. Conversely, the analysis revealed that factor like excessive iron and clay content were found to hinder yield predictability, emphasizing the need for targeted soil and management strategies. The fuzzy logic system proved effective for managing agricultural data uncertainties and variations that naturally occur.

The FOHEM model outperformed individual machine learning models such as RF, CatBoost, and ELM by achieving lower error rates such as MSE, MAE, RMSE and higher R^2^ values. The performance enhancement of FOHEM resulted from FIS and GA optimization which dynamically adjusted component weights to optimize adaptation across different treatment scenarios. A comparative analysis with existing studies revealed that FOHEM achieves approximately 5% higher R^2^, which proves its enhanced predictive accuracy and adaptability.

### 5.1 Practical implication and application

Beyond accuracy improvements, the FOHEM model holds significant practical implications and real world application potential. It functions as an advanced decision-support systems for optimizing intercropping strategies by predicting yield outcomes under varying conditions. By incorporating FIS, it effectively mitigates uncertainty and ensures reliable yield predictions even under fluctuating environmental conditions. The model facilitates precise decision-making regarding soil fertility, nutrient balance, and resource management, enabling efficient agricultural planning. Although FOHEM is not currently integrated with real time sensors, mobile applications, or cloud-based platforms, its architecture is lightweight enough to be adapted into desktop-based platforms. These could be used by agronomists, researchers, and extension workers for offline decision support in regions with limited connectivity or computing resources. With further development, FOHEM’s framework can be incorporated into mobile or web based applications to deliver actionable insights to farmers and policymakers, improving accessibility and facilitating evidence-based crop management. These advancements position FOHEM as a valuable tool for sustainable agriculture, supporting farmers and policymakers in improving yield efficiency and resource utilization.

### 5.2 Limitations and future work

Despite its advantages, the FOHEM model has certain limitations. One key limitation is its geographic and climatic scope, as the model was trained on data from semi-arid regions of Pakistan. Consequently, its generalization ability to other climatic zones such as tropical, temperate, or arid regions has not yet been validated. Environmental and soil characteristics in these zones can differ substantially, which may affect the model’s predictive accuracy.

Future research will involve expanding the dataset to include diverse agro-ecological zones and conducting cross-regional validation to ensure robust performance and broader applicability. Furthermore, future work will focus on integrating real-time data acquisition through sensor-based soil analysis. Deploying in-field sensors for parameters such as soil moisture, nutrient concentrations, pH, and temperature will allow the FOHEM model to operate on dynamic, real-world data streams, enhancing its prediction reliability and enabling more timely and site-specific decision support for farmers.

Moreover, the continued refinement of the fuzzy inference system will further strengthen decision-making capabilities under variable and uncertain environmental conditions. By addressing these limitations and advancing its real-time adaptability, the FOHEM model can evolve into a more scalable, practical, and impactful tool for optimizing intercropping systems and promoting sustainable agricultural practices.

## 6 Conclusion

This study reveals the proposed FOHEM model can accurately estimate yields of maize-soybean intercropping systems by integrating fuzzy logic; stacked ensemble learning and GA based weight optimization. By combining human expertise and data-driven learning, the model enhances predictive accuracy while maintaining interpretability and reliability. The model effectively captures yield dynamics across various intercropping treatments SS, SM, 2M2S and 2M3S, validating its adaptability and precision in supporting sustainable agricultural decision making. The dynamic weight optimization using GA, enables the system to automatically adjust contributions from each base learner, ensuring consistently improved predictions under varying conditions. The integration of LIME and SHAP, as state of the art explainability techniques, allows the model to be explainable by pointing out the features that have an impact on the yield prediction, thus improving the trust and transparency in the decision making process. The FOHEM model’s robust performance, evaluated by its lower error metrics MSE, MAE, RMSE and higher R^2^ values, validates its utility in handling complex agricultural data and making precise predictions across diverse treatment scenarios. This adaptability and accuracy make it a valuable decision support tool for farmers and policy makers, promoting sustainable agricultural practices through effective decision making.

## Data Availability

The raw data supporting the conclusions of this article will be made available by the authors, without undue reservation.
